# Biotyping and Genotyping (MLVA16) of *Brucella abortus* Isolated from Cattle in Brazil, 1977 to 2008

**DOI:** 10.1371/journal.pone.0081152

**Published:** 2013-12-06

**Authors:** Sílvia Minharro, Juliana P. Silva Mol, Elaine M. S. Dorneles, Rebeca B. Pauletti, Heinrich Neubauer, Falk Melzer, Fernando P. Poester, Maurício G. Dasso, Elaine S. Pinheiro, Paulo M. Soares Filho, Renato L. Santos, Marcos B. Heinemann, Andrey P. Lage

**Affiliations:** 1 Departamento de Medicina Veterinária Preventiva, Escola de Veterinária, Universidade Federal de Minas Gerais, Belo Horizonte, Minas Gerais, Brazil; 2 Escola de Medicina Veterinária e Zootecnia, Universidade Federal do Tocantins, Araguaína, Tocantins, Brazil; 3 Friedrich-Loeffler-Institut, Bundesforschungsinstitut für Tiergesundheit, Institut für bakterielle Infektionen und Zoonosen, Jena, Germany; 4 Fundação Estadual de Pesquisa Agropecuária, Instituto de Pesquisas Veterinárias Desidério Finamor, Eldorado do Sul, Rio Grande do Sul, Brazil; 5 Instituto Biológico, Centro de Pesquisa e Desenvolvimento de Sanidade Animal, São Paulo, São Paulo, Brazil; 6 Laboratório Nacional Agropecuário, Ministério da Agricultura, Pecuária e Abastecimento, Pedro Leopoldo, Minas Gerais, Brazil; 7 Departamento de Clínica e Cirurgia Veterinárias, Escola de Veterinária, Universidade Federal de Minas Gerais, Belo Horizonte, Minas Gerais, Brazil; Catalan Institute for Water Research (ICRA), Spain

## Abstract

Brucellosis is a worldwide distributed zoonosis that causes important economic losses to animal production. In Brazil, information on the distribution of biovars and genotypes of *Brucella* spp. is scarce or unavailable. This study aimed (*i*) to biotype and genotype 137 Brazilian cattle isolates (from 1977 to 2008) of *B. abortus* and (*ii*) to analyze their distribution. *B. abortus* biovars 1, 2 and 3 (subgroup 3b) were confirmed and biovars 4 and 6 were first described in Brazil. Genotyping by the panel 1 revealed two groups, one clustering around genotype 40 and another around genotype 28. Panels 2A and 2B disclosed a high diversity among Brazilian *B. abortus* strains. Eighty-nine genotypes were found by MLVA16. MLVA16 panel 1 and 2 showed geographic clustering of some genotypes. Biotyping and MLVA16 genotyping of Brazilian *B. abortus* isolates were useful to better understand the epidemiology of bovine brucellosis in the region.

## Introduction

Brucellosis is a worldwide distributed zoonosis, responsible for considerable economic losses due to abortion and culling of infected animals [1]. The genus *Brucella* is currently composed of ten species preferentially associated with different host: *B. abortus* (cattle), *B. canis* (canids), *B. ceti* (cetaceans), *B. melitensis* (sheep and goats), *B. microti* (*Microtus arvalis*), *B. neotomae* (*Neotoma lepida*), *B. ovis* (sheep), *B. pinipedialis* (pinnipeds), *B. suis* (pigs), and *B. inopinata* (man?) [2].

Bacteriological culture is considered the gold standard for brucellosis diagnosis. Due to its high specificity and ability to identify different species and biovars (bv), bacteriological culture is very important for epidemiological studies, allowing the determination of the way the disease spreads in a region and the possible sources of infection [3], [4].

*Brucella abortus* biovars have distinct geographic distributions [5] *Brucella abortus* bv1 and *B. abortus* bv2 are worldwide distributed, while *B. abortus* bv3 is found predominantly in Italy, India, Egypt, and Africa. *B. abortus* bv5 is most commonly found in Germany and United Kingdom [5], but has also been observed in France [6]. *B. abortus* biovars 4 and 6 have been reported on Mexico and France, though less frequently than bv1, 2, and 3 [6], [7]. *B. abortus* bv4 was also reported in Canada [8], Chile, Ecuador, and Cuba [9]. In India, *B. abortus* bv1 is the most frequent, followed by *B. abortus* bv3, although bv4, 6, and 9 were also found [10].

There have been just few studies concerning the identification of the biovars of *Brucella* spp. in Brazil describing *B. abortus* bv1, 2 and 3 and *B. suis* bv1 [9], [11], [12]. Also, *B. canis* and *B. ovis* were found infecting domestic animals [3]. *Brucella melitensis* was not reported in Brazil [3], [4].

Although the great homogeneity of the genus *Brucella* [13], conserved *loci* containing repeated sequences were found. Analysis of the number of repeats of these *loci* (Variable Number of Tandem Repeats – VNTR) is used as tool in the study of brucellosis [14]–[17]. Also, VNTR analysis of various *loci* (Multiple *Locus* VNTR Analysis – MLVA) is very important for investigations of infection by *Brucella* spp. and was applied to epidemiological control and surveillance of brucellosis [14], [17]–[22]. Moreover, this assay proved to be a valuable tool to determine relationships among *Brucella* isolates from different animal species and from humans, as well as for epidemiological trace-back studies [23], [24].

Thus, the aims of the present study were (*i*) to biotype and genotype Brazilian cattle *B. abortus* strains and (*ii*) to analyze their distribution to support the Programa Nacional de Controle e Erradicação de Brucelose e Tuberculose (PNCEBT) [25].

## Materials and Methods

### Bacterial Strains

**Figure 1:**
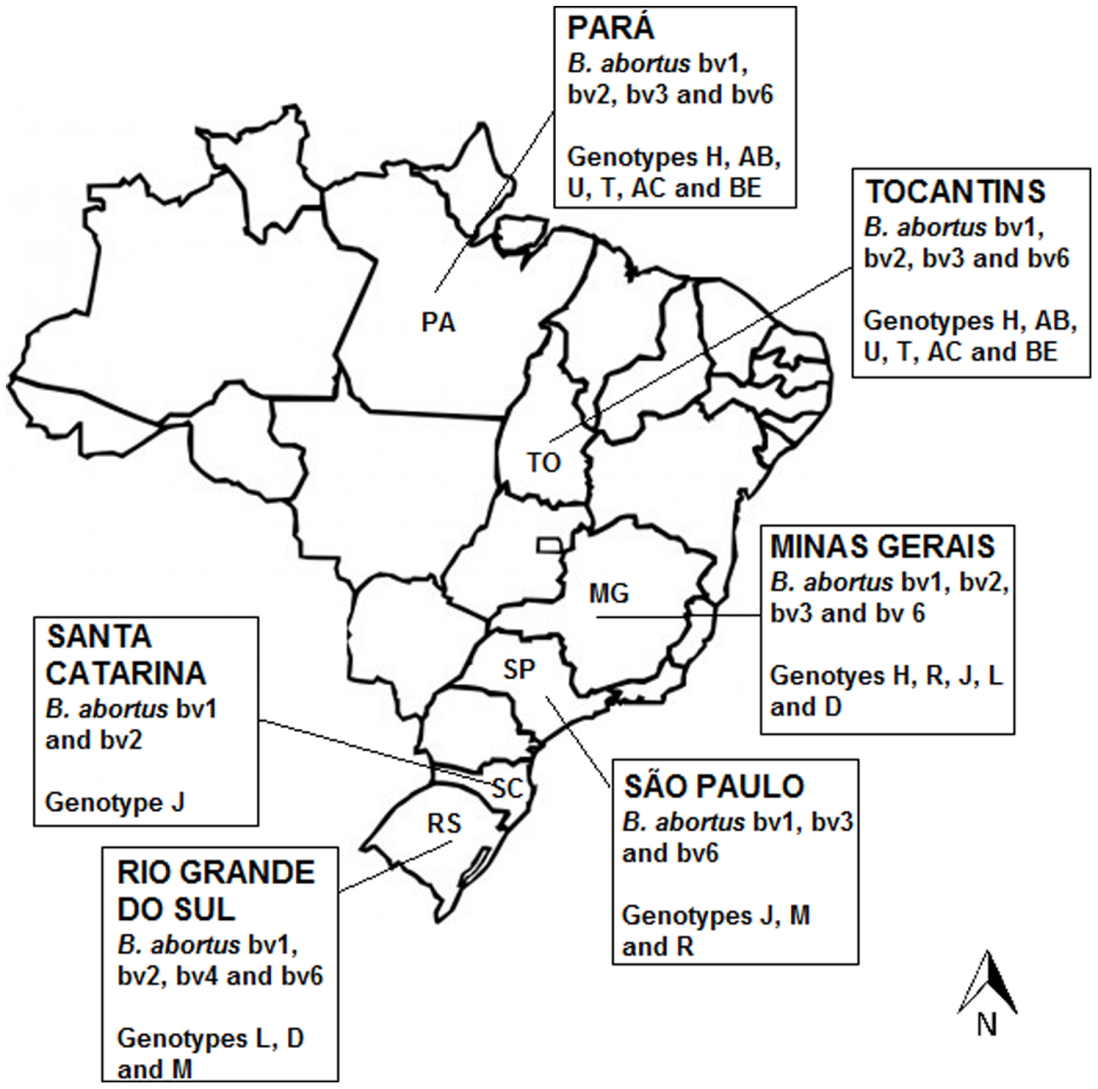
Sampling and distribution of biovars and MLVA16 Panel 2 genotypes of *B. abortus* isolates from Brazil, 1977 to 2008.

One hundred and thirty-seven Brazilian cattle *B. abortus* isolates, obtained from 1977 to 2008, were used. They were from the collections of Laboratório de Bacteriologia Aplicada, Escola de Veterinária, UFMG; Laboratório Nacional Agropecuário, Instituto de Pesquisas Veterinárias Desidério Finamor, and Instituto Biológico de São Paulo. These isolates were from Minas Gerais (38), Rio Grande do Sul (31), Pará (31), São Paulo (15), Tocantins (14) and Santa Catarina (8) (Figure 1). These strains represent the great majority of *B. abortus* isolates currently available in Brazil.

Reference strains, that were used as control in different procedures, were: *B. abortus* biovar (bv) 1 544 = ATCC 23448^T^, *B. abortus* bv2 ATCC 23449, *B. abortus* bv3 Tulya = ATCC 23450, *B. abortus* bv4 292 = ATCC 23451, *B. abortus* bv5 3196 = ATCC 23452, *B. abortus* bv6 870 = ATCC 23453 and *B. abortus* bv9 C68 = ATCC 23455, *B. abortus* bv1 B19 USDA, *B. abortus* bv1 RB51, *B. melitensis* bv1 16M = ATCC 23456^T^, *B. ovis* Reo 198, *B. suis* bv1 1330 = ATCC 23444, *Escherichia coli* ATCC 25922, *E. coli* B41, *Listeria monocytogenes* ATCC 19115, *Pseudomonas aeroginosa* ATCC 27853, *Salmonella enterica* biovar Typhimurium ATCC 14028 and *Staphylococcus aureus* ATCC 29213.

### Identification and Biotyping of B. abortus

All strains were identified by routine test [26], [27]. Isolates were also tested by *bcsp31*-PCR [28], AMOS-PCR [28], AMOS-ERY-PCR [30] for classification of subgroups 3a and 3b, and *omp2b*-RFLP-PCR with restriction by *Taq*I to confirm the identity of *B. abortus* bv4 strains [31].

### MLVA16 Genotyping

DNA from each strain was submitted to MLVA typing, using a subset of 16 tandem repeat *loci* (MLVA16) [15], [17].

### Genotype Analysis

Band size estimates were converted into number of repeat units for each *locus* [15], with the software BioNumerics 6.1 (Applied Maths, Sint-Martens-Latem, Belgium). Clustering analysis also was performed with the software BioNumerics 6.1 based on UPGMA [15]. The diversity index HGDI was calculated [32]. Genotypes obtained were compared to those deposited in the MLVAbank for Bacterial Genotyping (http://mlva.u-psud.fr/mlvav4/genotyping/index.php). The minimum spanning tree presented is the one with the highest overall reliability score and was calculated using UPGMA associated with the priority rule and the bootstrap resampling (BioNumerics 6.1).

## Results

### Biotyping and Identification of B. abortus Isolates

All the 137 isolates were confirmed to be *Brucella* spp. by *bcsp31*-PCR and to be *B. abortus* by biochemical tests, AMOS and AMOS-ERY-PCRs. Ninety-two (67.1%) of the 137 isolates were identified as *B. abortus* bv1, *B. abortus* bv2 or *B. abortus* bv4 by AMOS-PCR. These were found in all studied States, being bv1 most frequent in Rio Grande do Sul (26/72), followed by Minas Gerais (23/72), São Paulo (12/72), Santa Catarina (6/72), Pará (3/72) and Tocantins (2/72). *B. abortus* bv2 was most frequent in Minas Gerais (10/16), followed by Santa Catarina (2/16), Rio Grande do Sul (2/16), Pará (1/16) and Tocantins (1/16), while strains of bv3 were mainly observed in Pará (16/28), followed by Tocantins (9/28), Minas Gerais (2/28) and São Paulo (1/28). Similarly, bv6 strains were also more common in Pará (11/20), followed by Minas Gerais (3/20), Rio Grande do Sul (2/20), São Paulo (2/20) and Tocantins (2/20). Interestingly, only one strain of *B. abortus* bv4 was observed in Santa Catarina state. AMOS-PCR-negative strains were found in all States except Santa Catarina and most frequently in Pará and Tocantins.

*B. abortus* biovars found by biochemical testing were 1, 2, 3, 4, and 6. The most frequent was *B. abortus* bv1, followed by bv3, bv6, and bv2; the least frequent was bv4. The HGDI calculated for *B. abortus* biovar identification was 0.65. All *B. abortus* bv3 strains were classified as subgroup 3b by AMOS-ERY-PCR.

The unique *B. abortus* bv4 isolate, strain 16/02, isolated in Rio Grande Sul, was confirmed by *omp*2b-RFLP-PCR [31].

### MLVA16 Typing and Clustering of B. abortus Isolates

MLVA16 panel 1 identified nine genotypes (Table 1), four (28, 32, 33 and 40) previously described [15] and five new ones (I to V). These genotypes had one or more *loci* of panel 1 minisatellites with a number of repeat units different from reported ones [15]. Among genotypes observed for panel 1 genotyping the most frequent was genotype 28 found in all studied states, being more common in Minas Gerais (33), followed by Rio Grande do Sul (27/82), São Paulo (9/82), Santa Catarina (8/82), Tocantins (3/82) and Pará (2/82). The genotype 40 was more observed in Pará (26/45), followed by Tocantins (9/45), Minas Gerais (5/45), São Paulo (3/45) and Rio Grande do Sul (2/45). Whereas, the three strains of genotype 33 were observed in Tocantins (2/3) and São Paulo (1/3) and genotype 32 was found only in Rio Grande do Sul (1/1). Regarding new genotypes of panel 1, the profile I and II were observed only in Tocantins state, while patterns III and IV were found respectively in Pará and Rio Grande do Sul, all with one representative each. With two representative, genotype V was observed only in São Paulo state. The HGDI for panel 1 genotyping was 0.54.

**Table 1:** Biotyping, AMOS-PCR and genotyping based on panel 1 of MLVA16 of the strains of *B. abortus* isolated from cattle in Brazil, 1977 to 2008.

		Genotype^a^									
Biovar	AMOS-PCR	28 (%)	32 (%)	33 (%)	40 (%)	I (%)	II (%)	III (%)	IV (%)	V (%)	Total (%)
1	Pos	67 (48.9)	–	2 (1.5)	–	–	–	–	1 (0.7)	2 (1.5)	72 (52.6)
	Neg	–b	–	–	–	–	–	–	–	–	0 (0.0)
2	Pos	15 (10.9)	–	1 (0.7)	–	–	–	–	–	–	16 (11.7)
	Neg	–	–	–	–	–	–	–	–	–	0 (0.0)
3	Pos	–	–	–	**3 (2.2)** c	–	–	–	–	–	3 (2.2)
	Neg	–	–	–	23 (16.8)	–	1 (0.7)	1 (0.7)	–	–	25 (18.6)
4	Pos	–	1 (0.7)	–	–	–	–	–	–	–	1 (0.7)
	Neg	–	–	–	–	–	–	–	–	–	0 (0.0)
6	Pos	–	–	–	–	–	–	–	–	–	0 (0.0)
	Neg	–	–	–	19 (13.9)	1 (0.7)	–	–	–	–	20 (14.6)
Total	–	82 (59.9)	1 (0.7)	3 (2.2)	45 (32.8)	1 (0.7)	1 (0.7)	1 (0.7)	1 (0.7)	2 (1.5)	137 (100.0)

a Panel 1 of MLVA16 is based on *loci* Bruce06, Bruce08, Bruce11, Bruce12, Bruce42, Bruce43 and Bruce55 [15].

b n = 0.

c result not expected.

Panel 2A identified 13 genotypes, five (8, 30, 32, 33 and 34) previously described [15] and eight new ones (2A1 to 2A8). Panel 2A HGDI was 0.74. Genotype 33 was the most frequent (62/137), followed by 32 (28/137), 34 (13/137), 8 (10/137) and 30 (8/137). Among newly described genotypes, 2A3 was the most frequent (4/137), followed by 2A2 and 2A8 (3/137).

In panel 2B, 63 different genotypes were found, 13 of which had been previously described (18, 29, 30, 45, 52, 53, 58, 64, 66, 87, 88, 90 and 91) and 50 new genotypes, identified as 2B1 to 2B50. The HGDI for these *loci* was 0.97. Among these genotypes, the most frequent was genotype 91 (11/137), followed by 19 (9/137), 4 (8/137), 5 (6/137), 18 and 8 (5/137). The genotypes 30, 53, 17, 22, and 48 were found each in four of the 137 strains and genotypes 52, 58, 90, 16, 30, 44 and 46 in three of them.

Table 2 shows HGDI for the 16 *loci* based on biovars of studied *B. abortus* strains. Panel 1 had HGDIs below 0.16. Panel 2A *loci* with higher diversity were Bruce19 and Bruce21. HGDIs for *B. abortus* bv2 (0.74) and bv6 (0.73) were the highest in panel 2A. Panel 2B had the highest HGDI among all biovars (0.93 to 0.96); with *B. abortus* bv1 presenting the highest one. HGDI for MLVA16 (panels 1, 2A and 2B) was 0.98, differentiating 89 genotypes among the 137 studied strains (A to CY).

**Table 2:** Diversity index (HGDI) calculated for each *locus* and for panels 1, 2A, 2B and MLVA16 according to the biovars of *B. abortus* isolated from cattle, Brazil, 1977 to 2008.

Group/*Locus*	Biovar 1		Biovar 2		Biovar 3		Biovar 6	
	Types^a^	HGDI^b^	Types	HGDI	Types	HGDI	Types	HGDI
Panel 1	5	0.16	2	0.07	3	0.14	2	0.1
Bruce06	2	0.05	2	0.12	2	0.07	2	0.09
Bruce08	2	0.03	1	0.00	2	0.07	1	0.00
Bruce11	2	0.03	1	0.00	1	0.00	2	0.09
Bruce12	3	0.08	1	0.00	1	0.00	2	0.09
Bruce42	1	0.00	1	0.00	1	0.00	1	0.00
Bruce43	1	0.00	1	0.00	1	0.00	1	0.00
Bruce45	2	0.03	1	0.00	1	0.00	2	0.09
Bruce55	1	0.00	1	0.00	2	0.07	1	0.00
Panel 2A	7	0.42	4	0.74	4	0.62	7	0.73
Bruce18	3	0.18	1	0.00	1	0.00	4	0.27
Bruce19	2	0.13	2	0.50	3	0.37	3	0.40
Bruce21	2	0.26	3	0.62	2	0.35	5	0.54
Panel 2B	38	0.96	10	0.93	18	0.95	12	0.95
Bruce04	4	0.49	2	0.50	9	0.88	8	0.87
Bruce07	3	0.46	2	0.12	2	0.07	3	0.33
Bruce09	4	0.08	1	0.00	2	0.07	2	0.09
Bruce16	6	0.70	3	0.59	2	0.35	2	0.18
Bruce30	6	0.62	4	0.67	3	0.20	2	0.18
MLVA16	48	0.93	14	0.98	21	0.97	16	0.98

a Number of genotypes;

b Diversity index calculated according to Hunter and Gaston (1988) [32].

**Figure 2:**
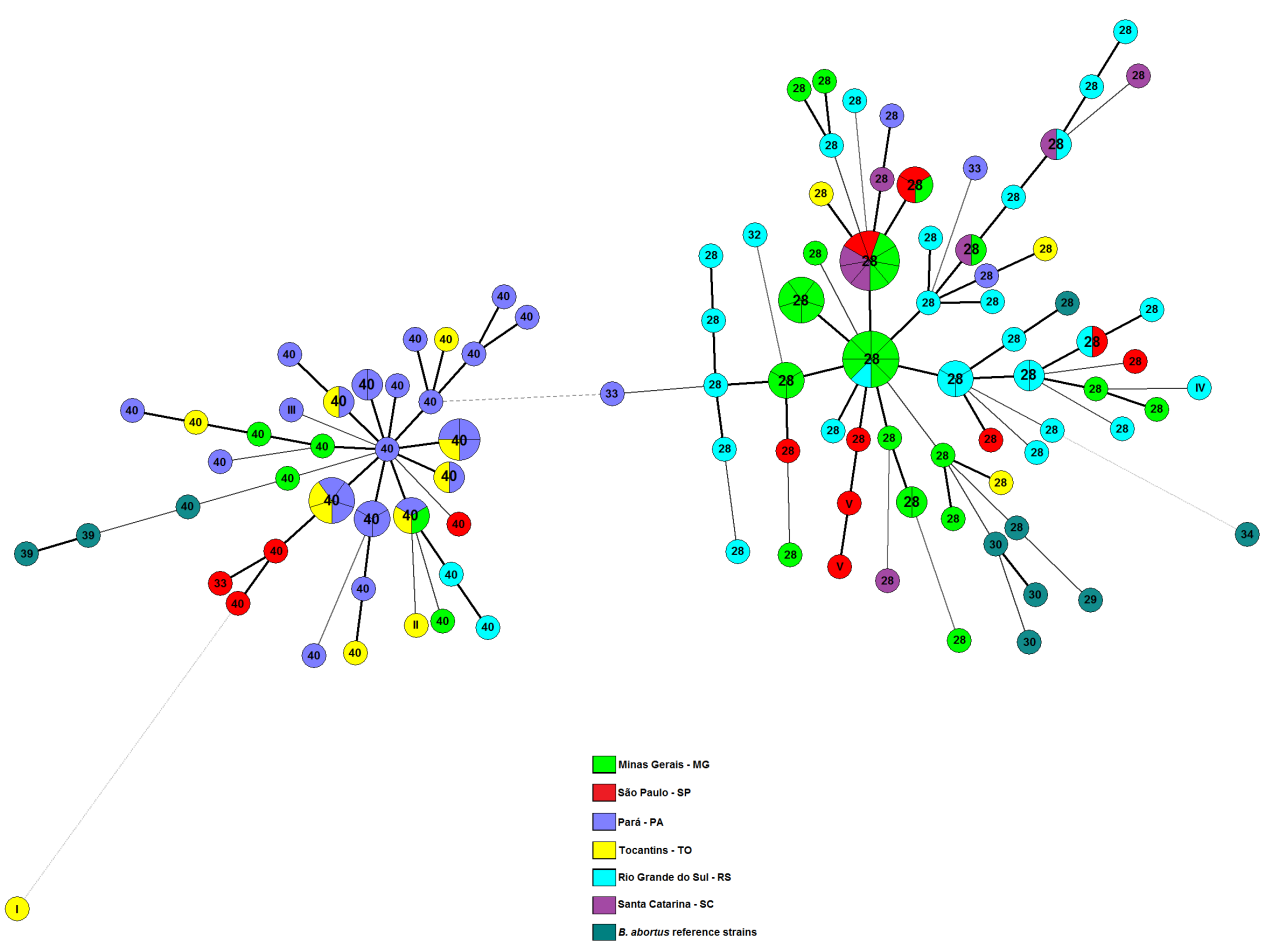
Minimum Spanning Tree (MST) analysis of *B. abortus* isolates using the MLVA16 data. The minimum spanning tree presented is the one with the highest overall reliability score and was calculated using UPGMA associated with the priority rule and the bootstrap resampling for strains of *Brucella abortus* isolated from cattle in Brazil, 1977 to 2008, the reference strains for each biovar of *B. abortus* and the vaccine strains S19 and RB51. Numbers in each clonal complex represent the genotype on Panel 1. Information on the origin of the isolates was color labeled.

The generated Minimum Spanning Tree (MST), using categorical and parsimonious coefficients, clearly separated the two groups (Figure 2).

Among MLVA16 genotypes found, the most frequent was genotype J (9/137; 6.6%), found in Minas Gerais, São Paulo, and Santa Catarina. Other frequent genotypes were D (5.8%; 8/137), found in Minas Gerais and Rio Grande do Sul; AB (3.6%; 5/137) and U (2.9%; 4/137), both found in Pará and Tocantins; H and R (2.2%; 3/137) from Minas Gerais, Pará and Tocantins, and Minas Gerais and São Paulo, respectively. Genotypes T, AC and BE (1.5%; 2/137) were found in Pará and Tocantins, and genotypes L and M (2.2%) in Minas Gerais and Santa Catarina, and in Rio Grande do Sul and São Paulo, respectively. Table S1 summarize the genotypic profile in MLVA16, genotypes in panel 1, alleles in each MLVA16 *loci*, biovar and field information of 137 cattle *B. abortus* isolates.

All new genotypes found in this study were included in MLVAbank (http://mlva.u-psud.fr/mlvav4/genotyping/index.php) in a cooperative database called Brucella_Brazil.

### Comparison among Typing Methods

Comparison of biochemical test results with panel 1 genotyping and AMOS-PCR showed compatible results by the three techniques in 68.6% (94/137) of the cases (Table 1). Twenty-three strains (16.8%) biochemically classified as *B. abortus* bv3 were from genotype 40 and negative by AMOS-PCR. Three strains biochemically classified as *B. abortus* bv3 were positive by AMOS-PCR and also from genotype 40. One strain phenotypically identified as *B. abortus* bv4 was AMOS-PCR positive and from genotype 32. Five strains (10.9%) that were AMOS-PCR positive *B. abortus* bv2 were typed as genotype 28. Another AMOS-PCR positive *B. abortus* bv2 was from genotype 33.

## Discussion

*Brucella abortus* biovars found in this study were 1, 2, 3, 4, and 6 (Table 1), the latter two being reported for the first time in Brazil. *B. abortus* bv1 was the most frequent biovar isolated, as also described by previous studies in Brazil [3], [12], [33] and most regions of the world [4], [18]. Interestingly, our *B. abortus* bv2 isolates did not need addition of serum to culture medium for their isolation, as it is usually described for strains of this biovar [27]. This phenomenon was also observed for other *B. abortus* bv2 strains from Brazil [12]. Of the two biovars that were described for the first time in Brazil (bv4 and bv6), only one strain of *B. abortus* bv4 was isolated from Rio Grande do Sul. Conversely, *B. abortus* bv6 was observed in all the States sampled except Santa Catarina.

In our study, the high isolation frequency of *B. abortus* bv 1 and bv2 could be explained by the continuous importation of cattle, since the beginning of colonization [34], from regions where these two biovars are or were endemic [1], [4], [10]. As all isolates of *B. abortus* bv3 were from sub-group 3b, they were more closely related to *B. abortus* bv3 of European than of African origin [30], [35], which is in accordance to the origin of most cattle imported into Brazil, that came from Europe and India [10], [34].The unique *B. abortus* bv4 isolate were from Rio Grande do Sul, at the border with Argentina, where this biovar has already been described [4].

*B. abortus* bv6 was not previously reported in South America. The highest frequency of this biovar was found in Pará, where 55% of the isolates were of *B. abortus* bv6. Although the way this biovar was introduced into Brazil is not clear, *B. abortus* bv6 is one of the most frequent biovars of *B. abortus* in cattle in India, which is origin of the Zebu breeds that predominate in Pará and most of the country [10], [34].

Although MLVA16 could correctly group the strains by species of *Brucella*, it was not able to identify *Brucella* spp. biovars, probably due to the high rates of mutation at some *loci*. Therefore, this technique is not an adequate substitute for classic biovar identification [15], which is supported by this study, as three genotypes (28, 33 and 40) were found in *B. abortus* from more than one biovar (Table 1).

The most frequent genotype among the studied Brazilian *B. abortus* isolates was genotype 28, found in all six States. In Santa Catarina, it was the only genotype found among the eight isolates of *B. abortus*. Genotype 28, generally reported to be associated with strains of *B. abortus* bv1 and 4 from USA, Costa Rica, Switzerland, France, Italy, and Germany, was observed in strains of *B. abortus* bv1 and in almost all (15/16) of *B. abortus* bv2 strains examined.

Genotype 40, first reported from strains of *B. abortus* bv6 from Africa, was also very frequently found in Brazil and is the most common among strains of *B. abortus* bv6 (95.0%) and *B. abortus* bv3 (92.9%). This explains the geographic distribution of this genotype, as these two biovars were also the most common in Pará and Tocantins.

The unique *B. abortus* bv4 strain, isolated from Rio Grande do Sul, was from genotype 32, previously reported among German *B. abortus* bv1 strains.Genotype 33 strains, described in *B. abortus* bv1 strains from France, were observed in this study in *B. abortus* bv3 strains from Pará (2) and São Paulo (1). The lack of sound epidemiological data on those isolates precludes any deeper inference on the way this genotype circulates in Brazil.

Among the newly described panel 1 genotypes, the major differences of genotype I are the presence of 11 repeat units at *locus* Bruce12 and six repeat units at *locus* Bruce45, whereas genotype II had only one repeat unit at *locus* Bruce55. Genotype III had as leading feature the presence of just four repeat units at *locus* Bruce08 and genotype IV, observed in a *B. abortus* bv1 isolated from Rio Grande do Sul, was mainly characterized by four repeat units at *locus* Bruce45. Genotype V strains are characterized by the presence of 13 repeat units at *locus* Bruce12. All of these new genotypes were still represented by just one strain each, exception for genotype V, which was observed in two strains from São Paulo. Additional systematic studies in the regions where those genotypes were observed could help to understand their epidemiological importance.

The HGDI calculated for MLVA16 panel 1 (0.54) was lower than that calculated for biotyping (0.65), indicating a lower discriminatory efficacy of MLVA16 panel 1, due to the presence of more than one biovar in the same genotype by this panel (Table 1). The HGDI calculated for MLVA16 panel 2A (0.74) is similar to that found by Al Dahouk et al. (2007) [17], who analyzed 128 human isolates of *B. melitensis* and found that the mean diversity index in panel 2A was less than 0.75. This panel has mainly helped in the differentiation of strains of bv2 and 6 (Table 2). The high HGDI calculated for MLVA16 panel 2B, which has the most variable *loci*, with diversity indexes generally higher than 0.80, demonstrates that it can be used, together with MLVA16 panel 2A, for identifying strains associated with disease outbreaks [14], [15], [17]. Comparing HGDI for MLVA16 panels 2A and 2B calculated in the present study and those described in the literature [15], [17], [19]–[21] leads to the conclusion that these *loci* were effective for differentiating the Brazilian *B. abortus* isolates, especially when they are compared to biotyping.

Genotyping based on MLVA16 identified 89 previously undescribed genotypes (http://mlva.u-psud.fr/mlvav4/genotyping/index.php) among Brazilian *B. abortus* strains. The difference between the number of genotypes identified by MLVA16 panel 1 and by MLVA16 panel 2 is a consequence of the hypervariability of the *loci* in this second panel [15], [17].

The results of genotyping by MLVA16 show that the same genotypes tend to circulate in neighboring States, such as genotypes AB, U, T, AC, and BE in Pará and Tocantins in North Region, and genotypes J, D, L, and M in Minas Gerais, São Paulo, Santa Catarina, and Rio Grande do Sul in Southeast and South regions. However, some genotypes, as genotype H, found in North (Pará and Tocantins) and Southeast (Minas Gerais) regions, were present in distant and unrelated regions, thus requiring newer studies to clarify their epidemiology.

The MST (Figure 2) showed that the *B. abortus* isolates from six Brazilian States were displayed into two well-defined clusters, with 51.7% similarity. Cluster I included *B. abortus* strains of genotypes 40, 33 (2/3), II and III, distributed in five of the six States, except Santa Catarina. Cluster I also included reference strains of *B. abortus* bv5 3196, *B. abortus* bv6 870 and *B. abortus* bv9 C68. Cluster II included *B. abortus* strains from genotypes 28, IV, 33 (1/3) and V, as well as reference strains of *B. abortus* bv1 544, *B. abortus* bv2 86/8/59 and *B. abortus* bv4 292 and vaccine strains *B. abortus* bv1 S19 and *B. abortus* bv1 RB51. Clustering was performed basically from *locus* Bruce04, which was the most variable, followed by *locus* Bruce19 and then by *locus* Bruce30, first grouping strains with higher number of repeat units and then those that had fewer repeat units. Similarly, MLVA analysis of the Spanish human *B. melitensis* strains showed a high diversity of genotypes with *locus* Bruce04 featuring the highest HGDI [20].

Two strains appeared apart from the main clusters: *B. abortus* bv3 reference strain Tulya, isolated from Africa, which is MLVA16 genotype 207, and *B. abortus* bv6 strain 95, isolated from Tocantins, which is MLVA16 genotype AR. This shows that these strains are more distantly related to the other Brazilian *B. abortus* isolates (Figure S1). Likewise, this can be observed in MST (Figure 2), which also shows that strain Tulya is distantly linked to cluster II (gathered around genotype 28) and that strain 95 is distantly linked to cluster I (gathered around genotype 40).

Typing the strains by biotyping, genotyping by MLVA16 panel 1 and AMOS-PCR gave compatible result in the majority of the cases. Most of the genotypes that were observed in the present study and were not previously described in a particular biovar (Table 1) are probably due to small numbers of isolates from these biovars that were already analyzed by MLVA16, being the present ones new descriptions of these genotypes in different biovars.

*B. abortus* bv3 is a heterogeneous biovar, divided into subgroups 3a and 3b [15], [30] and the MST (Figure 2) depicts these differences. All Brazilian *B. abortus* bv3 strains were closer to *B. abortus* bv6 reference strain 870 than to the *B. abortus* bv3 reference strain Tulya, which did not cluster with other strains. These results agreed to published reports, which showed that strains of *B. abortus* bv3 subgroup 3b present a pattern similar to that of strains of *B. abortus* bv5, *B. abortus* bv6 and *B. abortus* bv9 [30].

Interestingly, three strains of *B. abortus* bv3, genotype 40, isolated from Pará, showed positive results in AMOS-PCR, which was described as able of detecting only biovars 1, 2, and 4 of *B. abortus* [29]. However, occasionally, atypical isolates of *Brucella* spp., that do not match identification tables and keys [36], has been described. Banai et al. (1990) [37] found *B. mellitensis* bv1 strains that grew with acid fuchsin and thionine in two regions of Israel, suggesting evolution of a new variant. Corbel (1991) [38] also found isolates of *B. mellintensis* in Italy, Kuwait, Saudi Arabia, Zimbabwe, India, and Germany that were sensitive to thionine (20 µg/mL). Garcia et al. (1988) [39] isolated atypical strains of *B. abortus* from seven infected cattle herds in Ontario (Canada) that were sensitive to thionine and fuchsin, similar to *B. abortus* bv2, but they agglutinated in anti-M serum, which is characteristic of *B. abortus* bv4.

When these atypical isolates appear, it is necessary to determine if their appearance is a reproducible phenomenon, indicating a local taxonomic pattern, and not a laboratory error. If reproducibility is proven, this can be used as an epidemiological marker [27]. Bricker (2002) [36] reported that due to the small number of differences among species and biovars of *Brucella* spp., the smallest mutation could result in conflicting data, making interpretation of the characterization of the isolates difficult. The three strains of *B. abortus* bv3, genotype 40, that were reactive in AMOS-PCR were isolated from the municipality of Conceição do Araguaia, Pará, within an interval of four years between the isolation of the first strain and the other two; which were isolated within an interval of five months. This could be an indication that these markers are characteristic of atypical strains from this region.

Biotyping and MLVA16 genotyping of Brazilian isolates of *B. abortus* will allow the construction of a database of genotypes of *Brucella* spp. from Brazil, and, in the future, for Latin America, which certainly will contribute to a better understanding of the epidemiology and control of bovine brucellosis in the region.

## Supporting Information

Figure S1 (TIF)

Table S1 (DOCX)
