# Supplementary figures and images for: Biotyping and Genotyping (MLVA16) of Brucella abortus Isolated from Cattle in Brazil, 1977 to 2008
Source: PLoS One. 2013 Dec 6;8(12):e81152. doi: 10.1371/journal.pone.0081152 (PMC3855697; doi:10.1371/journal.pone.0081152)

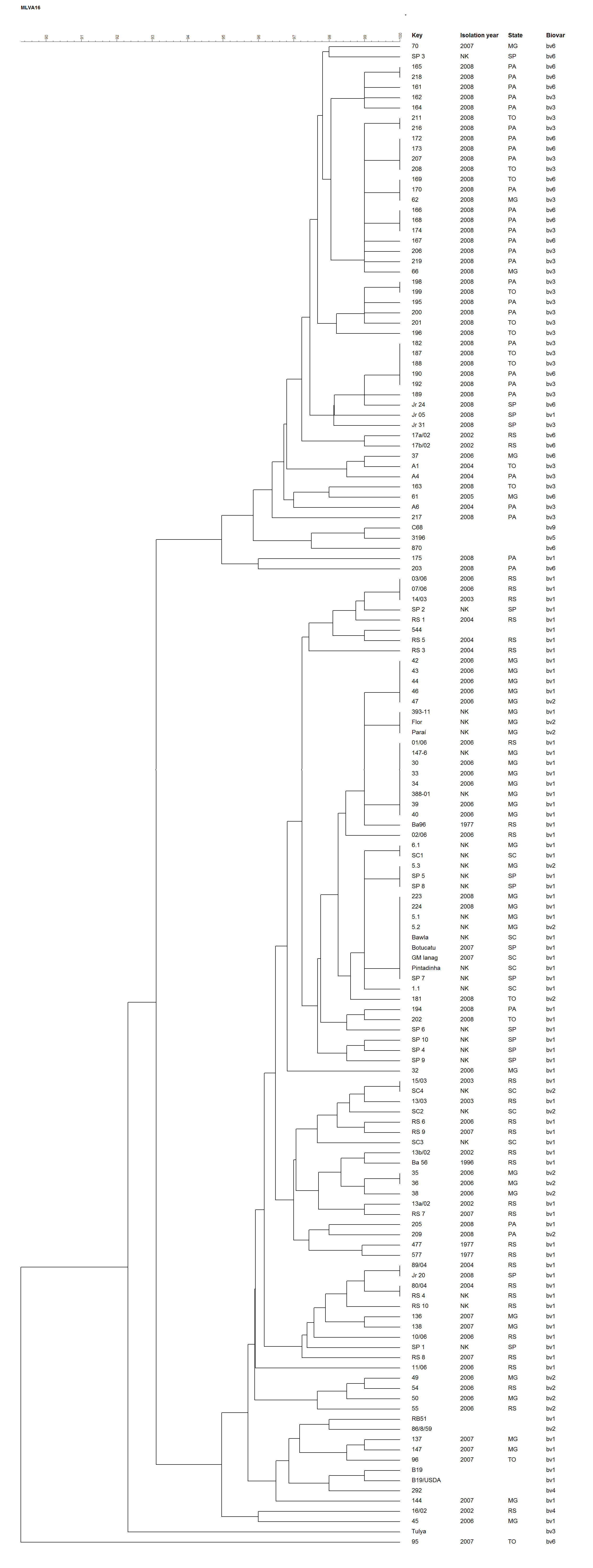

Supplement: Figure S1 — (TIF) [file pone.0081152.s001.tif]
