# Supplementary material for: Biotyping and Genotyping (MLVA16) of Brucella abortus Isolated from Cattle in Brazil, 1977 to 2008
Source: PLoS One. 2013 Dec 6;8(12):e81152. doi: 10.1371/journal.pone.0081152 (PMC3855697; doi:10.1371/journal.pone.0081152)
Supplement: Table S1 — (DOCX) [file pone.0081152.s002.docx]

Table S1 – Genotypic profile in MLVA16, genotypes in panel 1, alleles in each MLVA16 *loci*, biovar and field information of 137 cattle *Brucella abortus* isolates

| Strain | Biovar | State | Isolation  Year | Bruce | | | | | | | | | | | | | | | | Panel 1^a^ | MLVA16^b^ |
| --- | --- | --- | --- | --- | --- | --- | --- | --- | --- | --- | --- | --- | --- | --- | --- | --- | --- | --- | --- | --- | --- |
|  |  |  |  | 06 | 8 | 11 | 12 | 42 | 43 | 45 | 55 | 18 | 19 | 21 | 04 | 07 | 09 | 16 | 30 |  |  |
| A1 | bv3 | TO | 2004 | 3 | 5 | 3 | 12 | 2 | 2 | 3 | 3 | 7 | 48 | 8 | 9 | 4 | 3 | 3 | 4 | 40 | A |
| A4 | bv3 | PA | 2004 | 3 | 5 | 3 | 12 | 2 | 2 | 3 | 3 | 7 | 48 | 8 | 9 | 4 | 3 | 3 | 8 | 40 | B |
| A6 | bv3 | PA | 2004 | 3 | 5 | 3 | 12 | 2 | 2 | 3 | 3 | 7 | 46 | 8 | 9 | 4 | 7 | 3 | 3 | 40 | C |
| 30 | bv1 | MG | 2006 | 4 | 5 | 4 | 12 | 2 | 2 | 3 | 3 | 6 | 44 | 8 | 3 | 4 | 3 | 4 | 5 | 28 | D |
| 32 | bv1 | MG | 2006 | 4 | 5 | 4 | 12 | 2 | 2 | 3 | 3 | 7 | 44 | 8 | 3 | 4 | 5 | 4 | 5 | 28 | AF |
| 33 | bv1 | MG | 2006 | 4 | 5 | 4 | 12 | 2 | 2 | 3 | 3 | 6 | 44 | 8 | 3 | 4 | 3 | 4 | 5 | 28 | D |
| 34 | bv1 | MG | 2006 | 4 | 5 | 4 | 12 | 2 | 2 | 3 | 3 | 6 | 44 | 8 | 3 | 4 | 3 | 4 | 5 | 28 | D |
| 35 | bv2 | MG | 2006 | 4 | 5 | 4 | 12 | 2 | 2 | 3 | 3 | 6 | 46 | 8 | 4 | 4 | 3 | 4 | 5 | 28 | E |
| 36 | bv2 | MG | 2006 | 4 | 5 | 4 | 12 | 2 | 2 | 3 | 3 | 6 | 46 | 8 | 4 | 4 | 3 | 4 | 5 | 28 | E |
| 37 | bv6 | MG | 2006 | 3 | 5 | 3 | 12 | 2 | 2 | 3 | 3 | 7 | 44 | 8 | 9 | 4 | 3 | 3 | 4 | 40 | AG |
| 38 | bv2 | MG | 2006 | 4 | 5 | 4 | 12 | 2 | 2 | 3 | 3 | 6 | 44 | 8 | 4 | 4 | 3 | 4 | 5 | 28 | AH |
| 39 | bv1 | MG | 2006 | 4 | 5 | 4 | 12 | 2 | 2 | 3 | 3 | 6 | 44 | 8 | 3 | 4 | 3 | 4 | 5 | 28 | D |
| 40 | bv1 | MG | 2006 | 4 | 5 | 4 | 12 | 2 | 2 | 3 | 3 | 6 | 44 | 8 | 3 | 4 | 3 | 4 | 5 | 28 | D |
| 42 | bv1 | MG | 2006 | 4 | 5 | 4 | 12 | 2 | 2 | 3 | 3 | 6 | 44 | 8 | 3 | 4 | 3 | 4 | 4 | 28 | G |
| 43 | bv1 | MG | 2006 | 4 | 5 | 4 | 12 | 2 | 2 | 3 | 3 | 6 | 44 | 8 | 3 | 4 | 3 | 4 | 4 | 28 | G |
| 44 | bv1 | MG | 2006 | 4 | 5 | 4 | 12 | 2 | 2 | 3 | 3 | 6 | 44 | 8 | 3 | 4 | 3 | 4 | 4 | 28 | G |
| 45 | bv1 | MG | 2006 | 4 | 5 | 4 | 12 | 2 | 2 | 3 | 3 | 7 | 46 | 8 | 7 | 4 | 3 | 4 | 3 | 28 | AI |
| 46 | bv1 | MG | 2006 | 4 | 5 | 4 | 12 | 2 | 2 | 3 | 3 | 6 | 44 | 8 | 3 | 4 | 3 | 4 | 4 | 28 | G |
| 47 | bv2 | MG | 2006 | 4 | 5 | 4 | 12 | 2 | 2 | 3 | 3 | 6 | 44 | 8 | 3 | 4 | 3 | 4 | 4 | 28 | G |
| 49 | bv2 | MG | 2006 | 4 | 5 | 4 | 12 | 2 | 2 | 3 | 3 | 6 | 46 | 7 | 3 | 4 | 3 | 6 | 5 | 28 | AJ |
| 50 | bv2 | MG | 2006 | 4 | 5 | 4 | 12 | 2 | 2 | 3 | 3 | 6 | 46 | 7 | 3 | 4 | 3 | 5 | 4 | 28 | AL |
| 54 | bv2 | RS | 2006 | 4 | 5 | 4 | 12 | 2 | 2 | 3 | 3 | 6 | 46 | 7 | 3 | 4 | 3 | 5 | 5 | 28 | AM |
| 55 | bv2 | RS | 2006 | 4 | 5 | 4 | 12 | 2 | 2 | 3 | 3 | 6 | 46 | 7 | 3 | 5 | 3 | 4 | 5 | 28 | AN |
| 61 | bv6 | MG | 2005 | 3 | 5 | 3 | 12 | 2 | 2 | 3 | 3 | 7 | 46 | 7 | 7 | 4 | 3 | 3 | 3 | 40 | AO |
| 62 | bv3 | MG | 2008 | 3 | 5 | 3 | 12 | 2 | 2 | 3 | 3 | 7 | 44 | 8 | 7 | 4 | 3 | 3 | 3 | 40 | H |
| 66 | bv3 | MG | 2008 | 3 | 5 | 3 | 12 | 2 | 2 | 3 | 3 | 7 | 44 | 8 | 9 | 4 | 3 | 3 | 3 | 40 | AP |
| 70 | bv6 | MG | 2007 | 3 | 5 | 3 | 12 | 2 | 2 | 3 | 3 | 7 | 44 | 8 | 8 | 6 | 3 | 3 | 3 | 40 | AQ |
| 95 | bv6 | TO | 2007 | 4 | 5 | 3 | 11 | 2 | 2 | 6 | 3 | 4 | 40 | 10 | 8 | 5 | 4 | 8 | 4 | I | AR |
| 96 | bv1 | TO | 2007 | 4 | 5 | 4 | 12 | 2 | 2 | 3 | 3 | 6 | 44 | 8 | 5 | 6 | 3 | 3 | 5 | 28 | AS |
| 136 | bv1 | MG | 2007 | 4 | 5 | 4 | 12 | 2 | 2 | 3 | 3 | 6 | 44 | 8 | 4 | 5 | 3 | 3 | 5 | 28 | AT |
| 137 | bv1 | MG | 2007 | 4 | 5 | 4 | 12 | 2 | 2 | 3 | 3 | 6 | 44 | 8 | 3 | 6 | 3 | 3 | 5 | 28 | AU |
| 138 | bv1 | MG | 2007 | 4 | 5 | 4 | 12 | 2 | 2 | 3 | 3 | 6 | 46 | 8 | 4 | 5 | 3 | 3 | 5 | 28 | AV |
| 144 | bv1 | MG | 2007 | 4 | 5 | 4 | 12 | 2 | 2 | 3 | 3 | 6 | 46 | 8 | 3 | 5 | 3 | 3 | 6 | 28 | AK |
| 147 | bv1 | MG | 2007 | 4 | 5 | 4 | 12 | 2 | 2 | 3 | 3 | 6 | 46 | 8 | 3 | 6 | 3 | 3 | 5 | 28 | AX |
| 161 | bv6 | PA | 2008 | 3 | 5 | 3 | 12 | 2 | 2 | 3 | 3 | 7 | 46 | 8 | 4 | 4 | 3 | 3 | 3 | 40 | AY |
| 162 | bv3 | PA | 2008 | 3 | 5 | 3 | 12 | 2 | 2 | 3 | 3 | 7 | 46 | 8 | 8 | 4 | 3 | 3 | 3 | 40 | AW |
| 163 | bv3 | TO | 2008 | 3 | 5 | 3 | 12 | 2 | 2 | 3 | 1 | 7 | 46 | 8 | 7 | 4 | 3 | 3 | 3 | II | AZ |
| 164 | bv3 | PA | 2008 | 3 | 5 | 3 | 12 | 2 | 2 | 3 | 3 | 7 | 46 | 8 | 10 | 4 | 3 | 3 | 3 | 40 | BB |
| 165 | bv6 | PA | 2008 | 3 | 5 | 3 | 12 | 2 | 2 | 3 | 3 | 7 | 46 | 8 | 13 | 4 | 3 | 3 | 3 | 40 | X |
| 166 | bv6 | PA | 2008 | 3 | 5 | 3 | 12 | 2 | 2 | 3 | 3 | 7 | 44 | 8 | 5 | 4 | 3 | 3 | 3 | 40 | Y |
| 167 | bv6 | PA | 2008 | 3 | 5 | 3 | 12 | 2 | 2 | 3 | 3 | 7 | 44 | 8 | 12 | 4 | 3 | 3 | 3 | 40 | BC |
| 168 | bv6 | PA | 2008 | 3 | 5 | 3 | 12 | 2 | 2 | 3 | 3 | 7 | 44 | 8 | 5 | 4 | 3 | 3 | 3 | 40 | Y |
| 169 | bv6 | TO | 2008 | 3 | 5 | 3 | 12 | 2 | 2 | 3 | 3 | 7 | 44 | 8 | 7 | 4 | 3 | 3 | 3 | 40 | H |
| 170 | bv6 | PA | 2008 | 3 | 5 | 3 | 12 | 2 | 2 | 3 | 3 | 7 | 44 | 8 | 7 | 4 | 3 | 3 | 3 | 40 | H |
| 172 | bv6 | PA | 2008 | 3 | 5 | 3 | 12 | 2 | 2 | 3 | 3 | 7 | 44 | 8 | 6 | 4 | 3 | 3 | 3 | 40 | U |
| 173 | bv6 | PA | 2008 | 3 | 5 | 3 | 12 | 2 | 2 | 3 | 3 | 7 | 44 | 8 | 6 | 4 | 3 | 3 | 3 | 40 | U |
| 174 | bv3 | PA | 2008 | 3 | 5 | 3 | 12 | 2 | 2 | 3 | 3 | 7 | 44 | 8 | 5 | 4 | 3 | 3 | 3 | 40 | Y |
| 175 | bv1 | PA | 2008 | 3 | 5 | 4 | 12 | 2 | 2 | 3 | 3 | 6 | 44 | 8 | 5 | 4 | 3 | 8 | 7 | 33 | BD |
| 181 | bv2 | TO | 2008 | 4 | 5 | 4 | 12 | 2 | 2 | 3 | 3 | 6 | 44 | 9 | 3 | 4 | 3 | 5 | 5 | 28 | BE |
| 182 | bv3 | PA | 2008 | 3 | 5 | 3 | 12 | 2 | 2 | 3 | 3 | 7 | 44 | 9 | 13 | 4 | 3 | 3 | 3 | 40 | AB |
| 187 | bv3 | TO | 2008 | 3 | 5 | 3 | 12 | 2 | 2 | 3 | 3 | 7 | 44 | 9 | 13 | 4 | 3 | 3 | 3 | 40 | AB |
| 188 | bv3 | TO | 2008 | 3 | 5 | 3 | 12 | 2 | 2 | 3 | 3 | 7 | 44 | 9 | 13 | 4 | 3 | 3 | 3 | 40 | AB |
| 189 | bv3 | PA | 2008 | 3 | 5 | 3 | 12 | 2 | 2 | 3 | 3 | 7 | 44 | 9 | 4 | 4 | 3 | 3 | 3 | 40 | BF |
| 190 | bv6 | PA | 2008 | 3 | 5 | 3 | 12 | 2 | 2 | 3 | 3 | 7 | 44 | 9 | 13 | 4 | 3 | 3 | 3 | 40 | AB |
| 192 | bv3 | PA | 2008 | 3 | 5 | 3 | 12 | 2 | 2 | 3 | 3 | 7 | 44 | 9 | 13 | 4 | 3 | 3 | 3 | 40 | AB |
| 194 | bv1 | PA | 2008 | 4 | 5 | 4 | 12 | 2 | 2 | 3 | 3 | 6 | 44 | 8 | 3 | 4 | 3 | 3 | 7 | 28 | BE |
| 195 | bv3 | PA | 2008 | 3 | 5 | 3 | 12 | 2 | 2 | 3 | 3 | 7 | 44 | 8 | 5 | 4 | 3 | 4 | 3 | 40 | BG |
| 196 | bv3 | TO | 2008 | 3 | 5 | 3 | 12 | 2 | 2 | 3 | 3 | 7 | 44 | 8 | 5 | 2 | 3 | 4 | 3 | 40 | BH |
| 198 | bv3 | PA | 2008 | 3 | 5 | 3 | 12 | 2 | 2 | 3 | 3 | 7 | 44 | 8 | 13 | 4 | 3 | 4 | 3 | 40 | AC |
| 199 | bv3 | TO | 2008 | 3 | 5 | 3 | 12 | 2 | 2 | 3 | 3 | 7 | 44 | 8 | 13 | 4 | 3 | 4 | 3 | 40 | AC |
| 200 | bv3 | PA | 2008 | 3 | 5 | 3 | 12 | 2 | 2 | 3 | 3 | 7 | 44 | 8 | 14 | 4 | 3 | 4 | 3 | 40 | BI |
| 201 | bv3 | TO | 2008 | 3 | 5 | 3 | 12 | 2 | 2 | 3 | 3 | 7 | 44 | 8 | 4 | 4 | 3 | 4 | 3 | 40 | BJ |
| 202 | bv1 | TO | 2008 | 4 | 5 | 4 | 12 | 2 | 2 | 3 | 3 | 6 | 44 | 9 | 3 | 4 | 3 | 3 | 7 | 28 | BK |
| 203 | bv6 | PA | 2008 | 3 | 5 | 3 | 12 | 2 | 2 | 3 | 3 | 9 | 44 | 3 | 5 | 4 | 3 | 8 | 3 | 40 | BL |
| 205 | bv1 | PA | 2008 | 4 | 5 | 4 | 12 | 2 | 2 | 3 | 3 | 6 | 44 | 8 | 4 | 4 | 3 | 5 | 8 | 28 | BM |
| 206 | bv3 | PA | 2008 | 3 | 5 | 3 | 12 | 2 | 2 | 3 | 3 | 7 | 44 | 8 | 13 | 4 | 3 | 3 | 3 | 40 | V |
| 207 | bv3 | PA | 2008 | 3 | 5 | 3 | 12 | 2 | 2 | 3 | 3 | 7 | 44 | 8 | 6 | 4 | 3 | 3 | 3 | 40 | U |
| 208 | bv3 | TO | 2008 | 3 | 5 | 3 | 12 | 2 | 2 | 3 | 3 | 7 | 44 | 8 | 6 | 4 | 3 | 3 | 3 | 40 | U |
| 209 | bv2 | PA | 2008 | 3 | 5 | 4 | 12 | 2 | 2 | 3 | 3 | 6 | 44 | 8 | 4 | 4 | 3 | 5 | 6 | 33 | BN |
| 211 | bv3 | TO | 2008 | 3 | 5 | 3 | 12 | 2 | 2 | 3 | 3 | 7 | 44 | 8 | 14 | 4 | 3 | 3 | 3 | 40 | T |
| 216 | bv3 | PA | 2008 | 3 | 5 | 3 | 12 | 2 | 2 | 3 | 3 | 7 | 44 | 8 | 14 | 4 | 3 | 3 | 3 | 40 | T |
| 217 | bv3 | PA | 2008 | 4 | 4 | 3 | 12 | 2 | 2 | 3 | 3 | 7 | 44 | 8 | 13 | 4 | 3 | 3 | 3 | III | V |
| 218 | bv6 | PA | 2008 | 3 | 5 | 3 | 12 | 2 | 2 | 3 | 3 | 7 | 46 | 8 | 13 | 4 | 3 | 3 | 3 | 40 | X |
| 219 | bv3 | PA | 2008 | 3 | 5 | 3 | 12 | 2 | 2 | 3 | 3 | 7 | 44 | 8 | 4 | 4 | 3 | 3 | 3 | 40 | BO |
| 223 | bv1 | MG | 2008 | 4 | 5 | 4 | 12 | 2 | 2 | 3 | 3 | 6 | 44 | 8 | 3 | 4 | 3 | 5 | 5 | 28 | J |
| 224 | bv1 | MG | 2008 | 4 | 5 | 4 | 12 | 2 | 2 | 3 | 3 | 6 | 44 | 8 | 3 | 4 | 3 | 5 | 5 | 28 | J |
| 01/06 | bv1 | RS | 2006 | 4 | 5 | 4 | 12 | 2 | 2 | 3 | 3 | 6 | 44 | 8 | 3 | 4 | 3 | 4 | 5 | 28 | D |
| 02/06 | bv1 | RS | 2006 | 4 | 5 | 4 | 12 | 2 | 2 | 3 | 3 | 6 | 44 | 8 | 3 | 4 | 9 | 4 | 5 | 28 | BP |
| 03/06 | bv1 | RS | 2006 | 4 | 5 | 4 | 12 | 2 | 2 | 3 | 3 | 6 | 44 | 8 | 3 | 5 | 3 | 4 | 5 | 28 | S |
| 07/06 | bv1 | RS | 2006 | 4 | 5 | 4 | 12 | 2 | 2 | 3 | 3 | 6 | 44 | 8 | 3 | 5 | 3 | 4 | 5 | 28 | S |
| 10/06 | bv1 | RS | 2006 | 4 | 5 | 4 | 12 | 2 | 2 | 4 | 3 | 6 | 44 | 9 | 4 | 5 | 3 | 3 | 5 | IV | BQ |
| 11/06 | bv1 | RS | 2006 | 4 | 5 | 4 | 12 | 2 | 2 | 3 | 3 | 6 | 44 | 8 | 3 | 5 | 12 | 5 | 4 | 28 | BR |
| 13/03 | bv1 | RS | 2003 | 4 | 5 | 4 | 12 | 2 | 2 | 3 | 3 | 6 | 44 | 8 | 4 | 4 | 3 | 5 | 7 | 28 | BS |
| 13a/02 | bv1 | RS | 2002 | 4 | 5 | 4 | 12 | 2 | 2 | 3 | 3 | 6 | 46 | 9 | 4 | 4 | 3 | 4 | 6 | 28 | BT |
| 13b/02 | bv1 | RS | 2002 | 4 | 5 | 4 | 12 | 2 | 2 | 3 | 3 | 6 | 44 | 8 | 4 | 4 | 3 | 4 | 6 | 28 | BU |
| 14/03 | bv1 | RS | 2003 | 4 | 5 | 4 | 12 | 2 | 2 | 3 | 3 | 6 | 44 | 8 | 3 | 5 | 3 | 4 | 5 | 28 | S |
| 15/03 | bv1 | RS | 2003 | 4 | 5 | 4 | 12 | 2 | 2 | 3 | 3 | 6 | 44 | 9 | 4 | 4 | 3 | 5 | 7 | 28 | O |
| 16/02 | bv4 | RS | 2002 | 4 | 5 | 4 | 12 | 1 | 2 | 3 | 3 | 6 | 46 | 8 | 1 | 4 | 3 | 4 | 6 | 32 | BV |
| 17a/02 | bv6 | RS | 2002 | 3 | 5 | 3 | 12 | 2 | 2 | 3 | 3 | 7 | 44 | 9 | 7 | 5 | 3 | 3 | 3 | 40 | BY |
| 17b/02 | bv6 | RS | 2002 | 3 | 5 | 3 | 12 | 2 | 2 | 3 | 3 | 7 | 44 | 8 | 7 | 5 | 3 | 3 | 3 | 40 | BW |
| 80/04 | bv1 | RS | 2004 | 4 | 5 | 4 | 12 | 2 | 2 | 3 | 3 | 6 | 44 | 8 | 4 | 5 | 3 | 4 | 5 | 28 | N |
| 89/04 | bv1 | RS | 2004 | 4 | 5 | 4 | 12 | 2 | 2 | 3 | 3 | 6 | 44 | 9 | 4 | 5 | 3 | 4 | 5 | 28 | M |
| 477 | bv1 | RS | 1977 | 4 | 5 | 4 | 12 |  | 2 | 3 | 3 | 5 | 44 | 8 | 4 | 4 | 3 | 5 | 6 | 28 | BZ |
| 577 | bv1 | RS | 1977 | 4 | 5 | 4 | 12 | 2 | 2 | 3 | 3 | 5 | 44 | 8 | 4 | 4 | 3 | 4 | 6 | 28 | CA |
| Ba 56 | bv1 | RS | 1996 | 4 | 5 | 4 | 12 | 2 | 2 | 3 | 3 | 6 | 44 | 8 | 4 | 4 | 3 | 4 | 7 | 28 | CB |
| Ba96 | bv1 | RS | 1977 | 4 | 5 | 4 | 12 | 2 | 2 | 3 | 3 | 6 | 44 | 8 | 3 | 4 | 3 | 4 | 7 | 28 | CC |
| RS 1 | bv1 | RS | 2004 | 4 | 5 | 4 | 12 | 2 | 2 | 3 | 3 | 6 | 44 | 8 | 3 | 5 | 3 | 4 | 7 | 28 | CD |
| RS 3 | bv1 | RS | 2004 | 3 | 5 | 4 | 12 | 2 | 2 | 3 | 3 | 6 | 44 | 8 | 3 | 5 | 3 | 3 | 5 | 28 | CE |
| RS 4 | bv1 | RS | NK | 4 | 5 | 4 | 12 | 2 | 2 | 3 | 3 | 6 | 44 | 8 | 4 | 5 | 3 | 4 | 5 | 28 | N |
| RS 5 | bv1 | RS | 2004 | 4 | 5 | 4 | 12 | 2 | 2 | 3 | 3 | 5 | 44 | 8 | 3 | 5 | 3 | 4 | 5 | 28 | CF |
| RS 6 | bv1 | RS | 2006 | 4 | 5 | 4 | 12 | 2 | 2 | 3 | 3 | 6 | 44 | 9 | 4 | 4 | 3 | 5 | 4 | 28 | CG |
| RS 7 | bv1 | RS | 2007 | 4 | 5 | 4 | 12 | 2 | 2 | 3 | 3 | 6 | 44 | 9 | 4 | 4 | 3 | 4 | 6 | 28 | CH |
| RS 8 | bv1 | RS | 2007 | 4 | 5 | 4 | 12 | 2 | 2 | 3 | 3 | 6 | 44 | 8 | 4 | 5 | 4 | 6 | 5 | 28 | CI |
| RS 9 | bv1 | RS | 2007 | 4 | 5 | 4 | 12 | 2 | 2 | 3 | 3 | 6 | 44 | 9 | 4 | 4 | 3 | 6 | 4 | 28 | CJ |
| RS 10 | bv1 | RS | NK | 4 | 5 | 4 | 12 | 2 | 2 | 3 | 3 | 6 | 44 | 9 | 4 | 5 | 3 | 5 | 5 | 28 | CK |
| SP 1 | bv1 | SP | NK | 4 | 5 | 4 | 12 | 2 | 2 | 3 | 3 | 7 | 44 | 8 | 4 | 5 | 3 | 5 | 5 | 28 | CL |
| SP 2 | bv1 | SP | NK | 4 | 5 | 4 | 12 | 2 | 2 | 3 | 3 | 6 | 44 | 8 | 3 | 5 | 3 | 6 | 5 | 28 | CM |
| SP 3 | bv6 | SP | NK | 3 | 5 | 3 | 12 | 2 | 2 | 3 | 3 | 6 | 44 | 8 | 8 | 4 | 3 | 3 | 3 | 40 | CN |
| SP 4 | bv1 | SP | NK | 4 | 5 | 4 | 12 | 2 | 2 | 3 | 3 | 6 | 44 | 8 | 3 | 4 | 3 | 6 | 5 | 28 | AD |
| SP 5 | bv1 | SP | NK | 4 | 5 | 4 | 12 | 2 | 2 | 3 | 3 | 6 | 44 | 8 | 3 | 4 | 3 | 5 | 6 | 28 | R |
| SP 6 | bv1 | SP | NK | 4 | 5 | 4 | 12 | 2 | 2 | 3 | 3 | 6 | 44 | 8 | 3 | 4 | 3 | 3 | 6 | 28 | CO |
| SP 7 | bv1 | SP | NK | 4 | 5 | 4 | 12 | 2 | 2 | 3 | 3 | 6 | 44 | 8 | 3 | 4 | 3 | 5 | 5 | 28 | J |
| SP 8 | bv1 | SP | NK | 4 | 5 | 4 | 12 | 2 | 2 | 3 | 3 | 6 | 44 | 8 | 3 | 4 | 3 | 5 | 6 | 28 | R |
| SP 9 | bv1 | SP | NK | 4 | 5 | 4 | 13 | 2 | 2 | 3 | 3 | 6 | 44 | 8 | 3 | 4 | 3 | 6 | 6 | V | CQ |
| SP 10 | bv1 | SP | NK | 4 | 5 | 4 | 13 | 2 | 2 | 3 | 3 | 6 | 44 | 8 | 3 | 4 | 3 | 6 | 5 | V | AD |
| Botucatu | bv1 | SP | 2007 | 4 | 5 | 4 | 12 | 2 | 2 | 3 | 3 | 6 | 44 | 8 | 3 | 4 | 3 | 5 | 5 | 28 | J |
| GM lanag | bv1 | SC | 2007 | 4 | 5 | 4 | 12 | 2 | 2 | 3 | 3 | 6 | 44 | 8 | 3 | 4 | 3 | 5 | 5 | 28 | J |
| Pintadinha | bv1 | SC | NK | 4 | 5 | 4 | 12 | 2 | 2 | 3 | 3 | 6 | 44 | 8 | 3 | 4 | 3 | 5 | 5 | 28 | J |
| Paraí | bv2 | MG | NK | 4 | 5 | 4 | 12 | 2 | 2 | 3 | 3 | 6 | 44 | 8 | 3 | 4 | 3 | 4 | 6 | 28 | K |
| Bawla | bv1 | SC | NK | 4 | 5 | 4 | 12 | 2 | 2 | 3 | 3 | 6 | 44 | 8 | 3 | 4 | 3 | 5 | 5 | 28 | J |
| 1.1 | bv1 | SC | NK | 4 | 5 | 4 | 12 | 2 | 2 | 3 | 3 | 6 | 44 | 8 | 3 | 4 | 3 | 5 | 8 | 28 | CR |
| 5.1 | bv1 | MG | NK | 4 | 5 | 4 | 12 | 2 | 2 | 3 | 3 | 6 | 44 | 8 | 3 | 4 | 3 | 5 | 5 | 28 | J |
| 5.2 | bv2 | MG | NK | 4 | 5 | 4 | 12 | 2 | 2 | 3 | 3 | 6 | 44 | 8 | 3 | 4 | 3 | 5 | 5 | 28 | J |
| 5.3 | bv2 | MG | NK | 4 | 5 | 4 | 12 | 2 | 2 | 3 | 3 | 6 | 44 | 8 | 3 | 4 | 3 | 5 | 6 | 28 | R |
| 6.1 | bv1 | MG | NK | 4 | 5 | 4 | 12 | 2 | 2 | 3 | 3 | 6 | 44 | 8 | 3 | 4 | 3 | 5 | 7 | 28 | L |
| 388-01 | bv1 | MG | NK | 4 | 5 | 4 | 12 | 2 | 2 | 3 | 3 | 6 | 44 | 8 | 3 | 4 | 3 | 4 | 5 | 28 | D |
| 147-6 | bv1 | MG | NK | 4 | 5 | 4 | 12 | 2 | 2 | 3 | 3 | 6 | 44 | 8 | 3 | 4 | 3 | 4 | 5 | 28 | D |
| 393-11 | bv1 | MG | NK | 4 | 5 | 4 | 12 | 2 | 2 | 3 | 3 | 6 | 44 | 8 | 3 | 4 | 3 | 4 | 6 | 28 | K |
| Flor | bv2 | MG | NK | 4 | 5 | 4 | 12 | 2 | 2 | 3 | 3 | 6 | 44 | 8 | 3 | 4 | 3 | 4 | 6 | 28 | K |
| SC1 | bv1 | SC | NK | 4 | 5 | 4 | 12 | 2 | 2 | 3 | 3 | 6 | 44 | 8 | 3 | 4 | 3 | 5 | 7 | 28 | L |
| SC2 | bv2 | SC | NK | 4 | 5 | 4 | 12 | 2 | 2 |  | 3 | 6 | 44 | 9 | 4 | 4 | 3 | 5 | 6 | 28 | CS |
| SC3 | bv1 | SC | NK | 4 | 5 | 4 | 12 | 2 |  | 3 | 3 | 6 | 44 | 9 | 4 | 4 | 3 | 9 | 5 | 28 | CT |
| SC4 | bv2 | SC | NK | 4 | 5 | 4 | 12 | 2 | 2 | 3 | 3 | 6 | 44 | 9 | 4 | 4 | 3 | 5 | 7 | 28 | O |
| Jr 20 | bv1 | SP | 2008 | 4 | 5 | 4 | 12 | 2 | 2 | 3 | 3 | 6 | 44 | 9 | 4 | 5 | 3 | 4 | 5 | 28 | M |
| Jr 31 | bv3 | SP | 2008 | 3 | 5 | 3 | 12 | 2 | 2 | 3 | 3 | 7 | 44 | 9 | 8 | 4 | 3 | 3 | 4 | 40 | CY |
| Jr 24 | bv6 | SP | 2008 | 3 | 5 | 3 | 12 | 2 | 2 | 3 | 3 | 7 | 44 | 9 | 8 | 4 | 3 | 3 | 3 | 40 | I |
| Jr 05 | bv1 | SP | 2008 | 3 | 5 | 4 | 12 | 2 | 2 | 3 | 3 | 7 | 44 | 9 | 8 | 4 | 3 | 3 | 3 | 33 | I |

^a^Genotype in panel 1

^b^Genotype in MLVA16
